# Supplementary material for: A parsimonious neutral model suggests Neanderthal replacement was determined by migration and random species drift
Source: Nat Commun. 2017 Oct 31;8:1040. doi: 10.1038/s41467-017-01043-z (PMC5717005; doi:10.1038/s41467-017-01043-z)
Supplement: Supplementary file 1 — Supplementary Information [file 41467_2017_1043_MOESM1_ESM.pdf]

Supplementary Information for:

"A parsimonious neutral model suggests Neanderthal replacement was determined by migration and random species drift"

Oren Kolodny, Marcus W Feldman

Department of Biology, Stanford University, Stanford, CA 94305

Content

Supplementary Table 1: Examples of studies that attribute Neanderthal replacement to environmental factors or to a selection disadvantage

Supplementary Table 2: References for the discussion of introgression, transitional techno-complexes and Modern spread in the early Upper Paleolithic (UP)

Supplementary Note 1: Assessment of the duration of replacement in simulations and in the archaeological record, and their comparison

Calculation of the duration of replacement

Comparing empirical and simulated duration of replacements

The Number of times that each band territory switched hands between species

Supplementary Note 2: Unidirectional migration

Supplementary Note 3: Additional notes on population sizes, population structure, the Levant, and inter-species competition dynamics

Supplementary Note 4: The likelihood of finding evidence in the archaeological record for local recurrent species replacement

Supplementary Section References

Supplementary Table 1: Examples of studies that attribute Neanderthal replacement to environmental factors or to a selection disadvantage

Environmental factors	Refs. [1–7].
Diet and subsistence	Refs. [8–11].
Life history	Refs. [12,13].
Cognitive and cultural capacities	Refs. [14–23].

Supplementary Table 2: References for the discussion of introgression, transitional techno-complexes and Modern spread in the early Upper Paleolithic (UP)

Introgression dynamics of Neanderthals and Modern humans	Refs. [24–30].
Ahmarian, Aurignacian, and transitional techno-complexes	Refs.[31–35], and ref. [36] and the ensuing comments.
Early UP spread into novel regions	Refs. [37–42].

Supplementary Note 1: Assessment of the duration of replacement in simulations and in the archaeological record, and their comparison

Calculation of the duration of replacement. The period calculated in the simulations that is compared to the archaeologically-supported period of co-existence of the two species should take into account the time point at which species' co-existence is likely to be evident in the archaeological record ('coexistence', here and elsewhere, refers to contemporaneous habitation of parts of the same deme, i.e. of Europe and the Levant, or of Eurasia, not necessarily with full regional overlap). That is, the duration should not be the full duration of each model simulation, but the simulation period during which both species are likely to have a demonstrable presence in the form of archaeological findings that can be clearly associated with the identity of the species that produced them. We explored five among many possible methods of calculating species' coexistence, which correspond to Supplementary Fig. 1a-e and supplementary Fig. 2a-e. These are:

a. Species coexistence is defined as the period during which both species are present in the overall population at a frequency of above 10%, between the last crossings of this threshold by each of the species, i.e. from the last time that Moderns became more frequent than 10% of the population to the time in which Neanderthals dropped below 10% for the last time. This is the method used for the results in the main text.
b. Species coexistence is defined as the period following the last crossing of the 10% threshold by the Moderns.
c. Species coexistence is defined as the period following the first crossing of the 10% threshold by the Moderns.
d. Species coexistence is defined as the sum of periods in which Moderns comprised over 10% of the overall population.
e. Species coexistence is defined as the sum of periods during which each population – Moderns and Neanderthals – made up over 10% of the overall population.

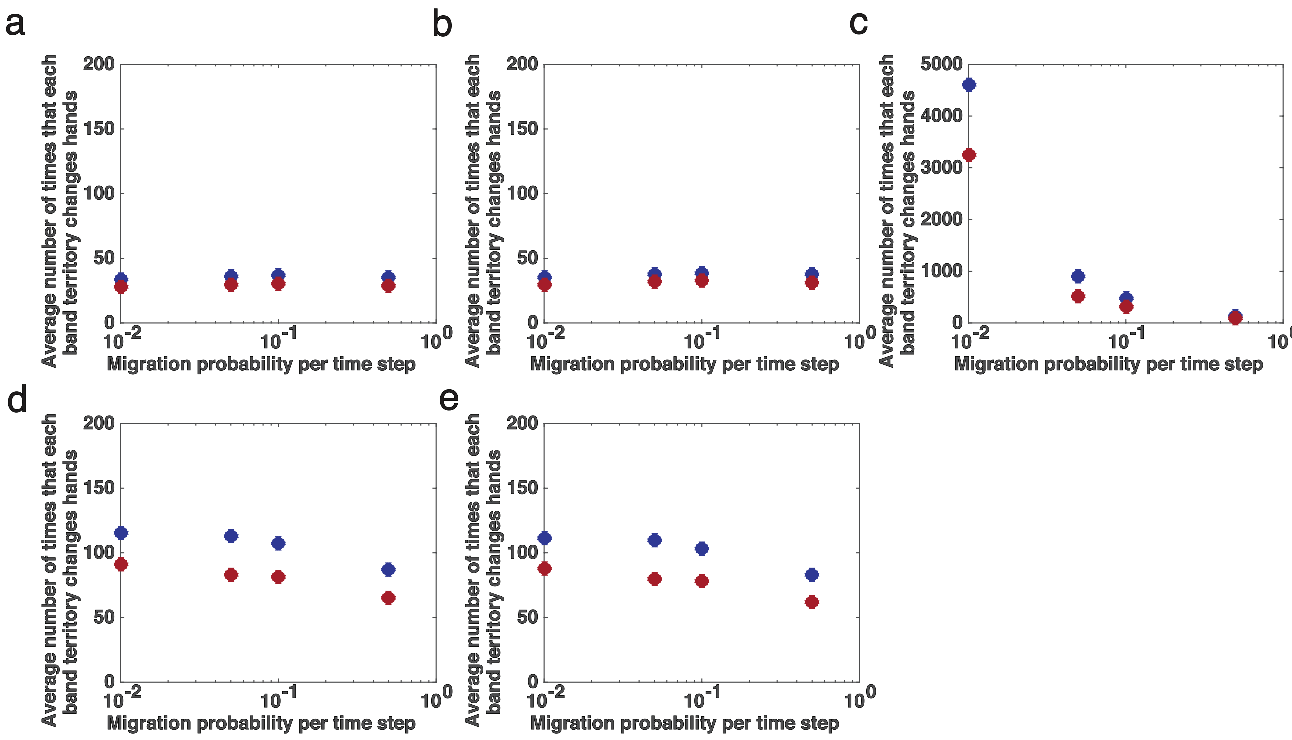

Supplementary Figure 1: Comparison of alternative methods for calculating species' coexistence.

The dark blue and dark red circles depict, respectively, the mean and the median average number of band replacement events per band territory during the process of species' replacement for various migration probabilities into Europe. Panels a-e correspond, respectively, to methods a-e for calculating the replacement duration from the time trajectories that are produced during the simulation. Europe's carrying capacity, N_I, is 50 bands. Note that the scale in panel c differs from that of the others.

Calculating the period of overlap between the two species should take into account such features of the archaeological record, as:

1. It is partial and patchy, both because the vast majority of their activities leave no archaeological record and because even the activities and artifacts that do have the potential to leave a record have an extremely high likelihood of being lost.

2. The vast majority of habitation sites have likely not been discovered, and most of them never will be.
3. In most sites there are no skeletal remains that can unambiguously associate the site or a locus in it with one *Homo* species and not the other.
4. A brief presence of a species at a certain site is unlikely to be correctly associated with it.
5. A particularly early or late presence of a species is likely to be missed or incorrectly classified because it is likely to be in a context with artifacts that are not recognized as typical of that species.

In choosing the method of coexistence calculation and the related threshold/s, points (4) and (5) are particularly important: an early and/or short-lived presence of Moderns in Europe is likely to be missed completely, as the remains expected from such a presence would be few and the cultural artifacts would be atypical of the culture that is associated with Moderns in later periods. In fact, the recent discovery of genetic introgression of Moderns into Neanderthals, which is estimated to have occurred much earlier than the first archaeological evidence of Moderns' presence in Europe, suggests that at least one period of coexistence had occurred for a non-negligible period of time and/or at non-negligible frequency, because such introgression is statistically unlikely to have been detected on the basis of few inter-species interactions. This suggests that methods such as (3) above should be rejected. Methods (4) and (5) are intermediates between method (3) and methods (1) and (2), as they take into account early occurrences, but under-weigh them compared to method (3). Their disadvantage is that their interpretation does not make much practical sense: from the earliest time point that a species' presence is established, unless it then disappears for millennia, a likely interpretation of the archaeological record would be that the species persisted with subsequent coexistence, even though in reality the species may have dropped below discovery threshold or have even gone extinct and later re-colonized. The difference between (1) and (2) is minor (see Supplementary Fig. 1a-b), and we chose method (1) for the sake of symmetry in the treatment of the two species. Note that methods (1), (2), (4) and (5) lead to similar results (Supplementary Fig. 2).

The chosen threshold of 10% is fairly arbitrary: we did not attempt to assess quantitatively the exact threshold of discovery that the considerations outlined above would suggest, and instead chose this threshold as a conservative value.

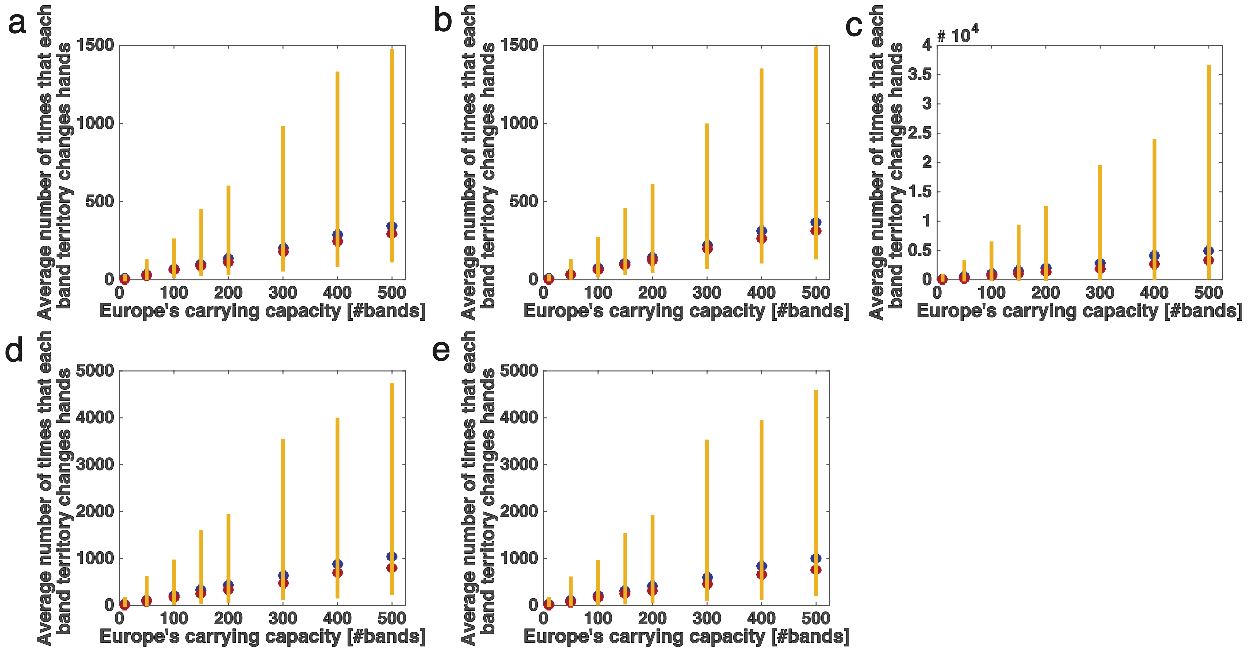

Supplementary Figure 2: Comparison of the replacement duration with different methods for calculation of species' coexistence.

The mean (dark blue circles) and median (orange circles) average number of band replacement events per band territory during the process of species' replacement for various carrying capacities in Europe. The range that covers 95% of simulation runs (n=500) is marked in dark yellow. Panels a-e correspond to methods a-e of calculating the replacement duration from the time trajectories that are produced during the simulation.

Comparing empirical and simulated duration of replacements. In the main text, Figure 2b shows the ranges of parameter combinations for which our null model should be rejected in a standard hypothesis test, under the assumption that the archaeologically-supported period of Moderns' and Neanderthals' coexistence in the Middle East and Europe is 12,000 years. This assumption for the duration of coexistence draws upon findings reported in multiple studies (see refs. [43–53]). Two observations among these that should be noted are the remains

of Moderns dated as early as 54,000 BP that have been unearthed in the middle east, in present-day Israel, a geographic range in which Neanderthals were known to exist between 60-40ka [48], providing an early bound for the two species' coexistence, and the finding that Neanderthals existed in Europe at least as late as 40ka [54]. Accordingly, the two species seem to have coexisted in parts of their ranges for at least 14,000 years and perhaps longer. We chose a period of 12,000 years of coexistence as a conservative estimate.

Alternative interpretation of some of the archaeological findings may lead to different estimates of the period of the two species' co-existence within the same geographical range. If one considers only Central and Western Europe as the zone of interaction between the two species, the duration of estimated overlap is significantly shorter, on the order of a few millennia [54]. Supplementary Figure 3b depicts the ranges of parameters for which our null model should be rejected in a standard hypothesis test at a significance level with p-value smaller than 0.05, assuming a period of species co-existence of 5000 years. As expected, the range for which the model should be rejected is wider than that suggested in the main text, but importantly, even assuming this shorter period of species overlap, wide ranges of possible replacement rates and population carrying capacities exist for which the model cannot be rejected.

At the other end of the spectrum of the estimates of duration of species' overlap, one may accept at face value the multiple findings of anatomically modern humans in the Levant during thousands of years, long before their spread into Europe, between approximately 140,000BP to 80,000BP. These may have been contemporaneous with Neanderthal populations in the region, which have been found there during part of this time span [52]. The cultures of the two species as reflected in the lithic record in the middle Paleolithic Levant are indistinguishable from one another, and the few sites from this period in which distinguishing skeletal remains are found, make it hard to definitively assess the demographic and ecological relationship between the two species (see discussion in ref. [52]), but it is plausible that the two species co-existed in parts of the Levant for long periods of time, with bands within and between the two species presumably competing with one another. The range of parameters for which our model should be rejected under this reconstruction of the species' history is smaller than that depicted in Figure 2b in the main text, and decreases as the estimated duration of species' coexistence in the Levant increases. Notably, a trajectory

along which the two species increase and decrease alternately in their frequencies over long periods of time is in line with our null model but does not support strong selection in favor of either species. This topic is elaborated in the main text's Discussion.

Our non-spatial model assumes within-deme panmixia, an obvious simplification of the realistic dynamics, in which geo-spatial structure may play a meaningful role (this is discussed in the main text and in Supplementary Note 3, and is further explored in the simple model that incorporates spatial structure). The within-deme panmixia in our model dictates that the duration of overlap that is estimated from the archaeological record and compared to our results take into account the full time span along which interactions between the two species take place within the deme, although it is likely that at any given moment, inter-species interactions were taking place only in few localities.

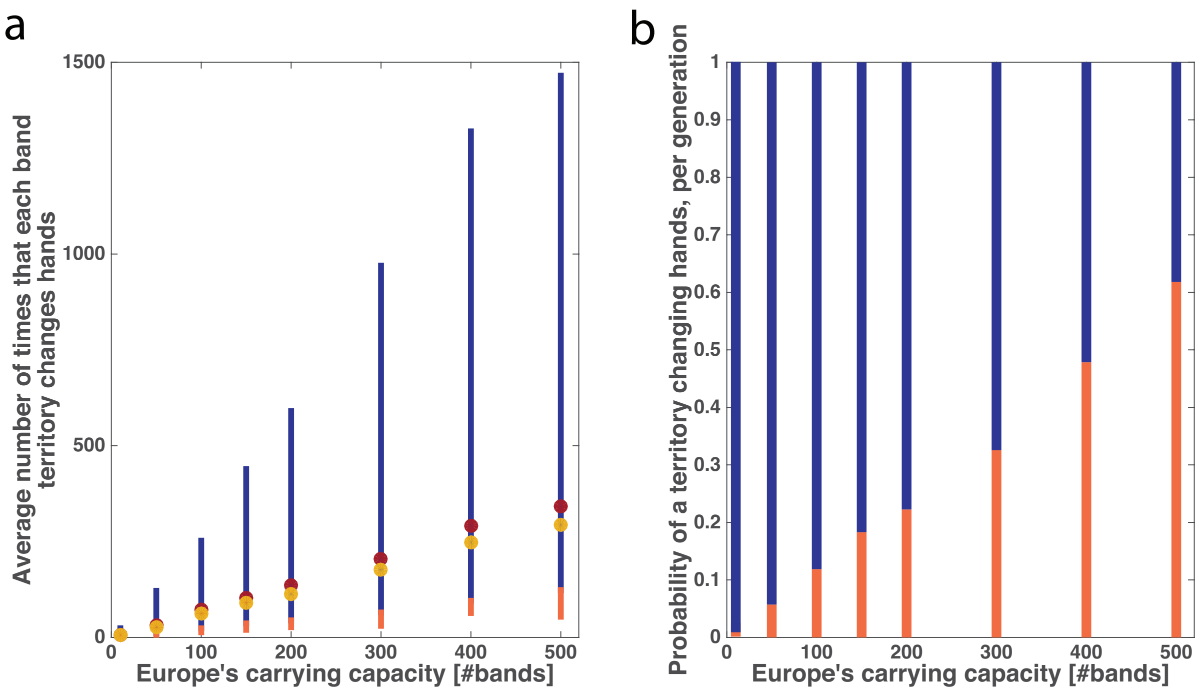

Supplementary Figure 3: Comparing model predictions to the archaeological record, assuming 5,000 years of species' overlap

(a) This panel is identical to Figure 2a in the main text. It plots the range of durations of species' coexistence in simulations with various carrying capacities in Europe, in units of average number of events of band replacement per band territory. The range that covers 95% of the results is marked in dark blue, and the 5% of the simulations with the shortest

coexistence durations is marked in orange (500 simulation replicates were conducted for each carrying capacity). The mean and median values are marked in brown and dark yellow, respectively. Coexistence duration is defined as the period during which both species segregate in the population at frequencies above 10%, between the last crossings of this threshold by each of the species, as demonstrated by the orange and yellow circles in the time trajectories of Figure 3. (b) Tests of the hypothesis of neutral replacement for a range of parameter combinations, assuming a species coexistence duration of 5,000 years: each point in the panel represents the result of a test at a significance level of α=0.05. The range of parameters for which neutral replacement should be rejected is denoted in orange. The range for which the model could not be rejected is marked in dark blue.

The number of times that each band territory switched hands between species. The previous section (Supplementary Fig. 3) and the main text (Fig. 2a) present the duration of species replacement, under the model presented in the main text, in terms of the mean number of times that each band territory changed hands; these changes of hands include both cases in which a band died out and was replaced by another band of the same species, and cases in which it was replaced by a band of the other species. It is interesting to derive from the simulations the breakdown of these two processes. Supplementary Fig. 4 depicts the mean numbers of inter-species replacements per site. On average, the number of inter-species replacement constitutes 39% of all replacements, for all values of the Carrying capacity of Europe. Although replacements are neutral, and once a band dies any of the remaining bands may replace it with equal probability, the majority of band replacements are within-species. This is because at any given moment one of the two species is a majority in the population, and it is both more likely that a majority-species band would be chosen to die out, and that a majority-species band would be chosen to replace it, giving rise to more within-species than inter-species replacements. Although they are a minority of band-territory replacements, inter-species replacements constitute a large fraction of the replacements. We suggest that this relatively high fraction is unrealistically high, and is an artifact of the major simplification in our model, i.e. that it is not spatially explicit. In a spatially explicit model, we expect that throughout the majority of the species' co-existence they would be distributed non-randomly, in such a way that neighboring bands are more likely than random to be of the

same species and also more likely to replace one another when one dies out. This would decrease significantly the fraction of the inter-species replacements of the total number of band replacements. This prediction is supported by the results of the simple spatially explicit model that we implemented (Fig. 6 in the main text).

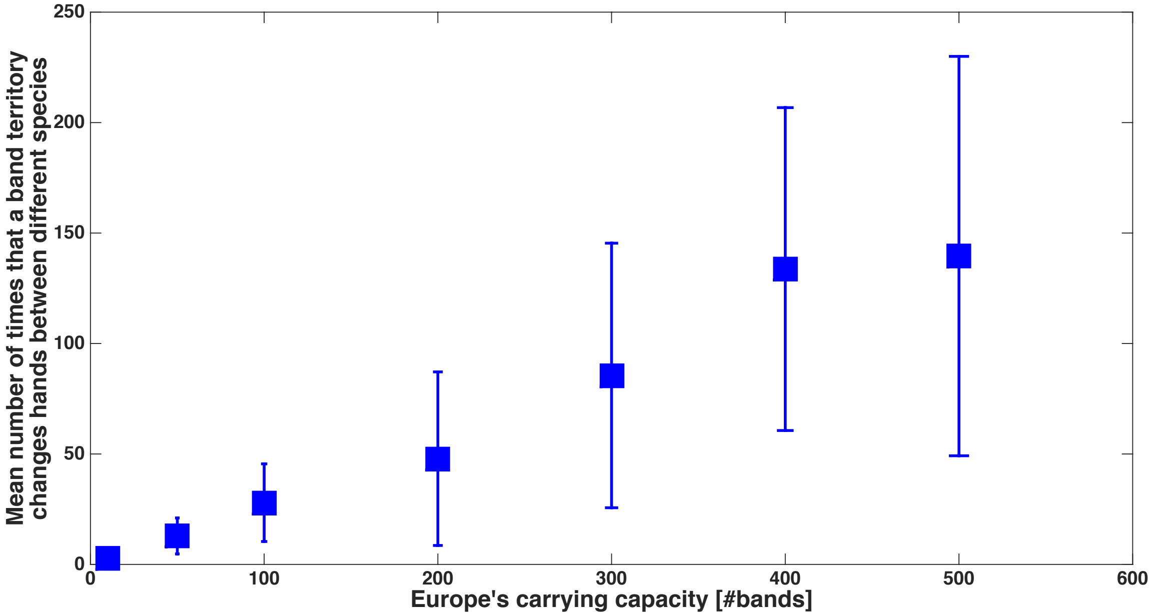

Supplementary Figure 4: The mean number of inter-species replacements that take place during the species replacement process. The error bars denote one standard deviation.

Supplementary Note 2: Unidirectional migration

The findings presented in the main text regarding the condition of unidirectional migration out of Africa reflect the results for a probability of migration into Europe per time step of $M_2 = 0.1$, and $N_2 = 100$ ($m_2 = 0.001$). Here we provide analogous results, for higher and for lower probabilities of migration (Supplementary Fig. 5). We find that these lead to qualitatively similar outcomes.

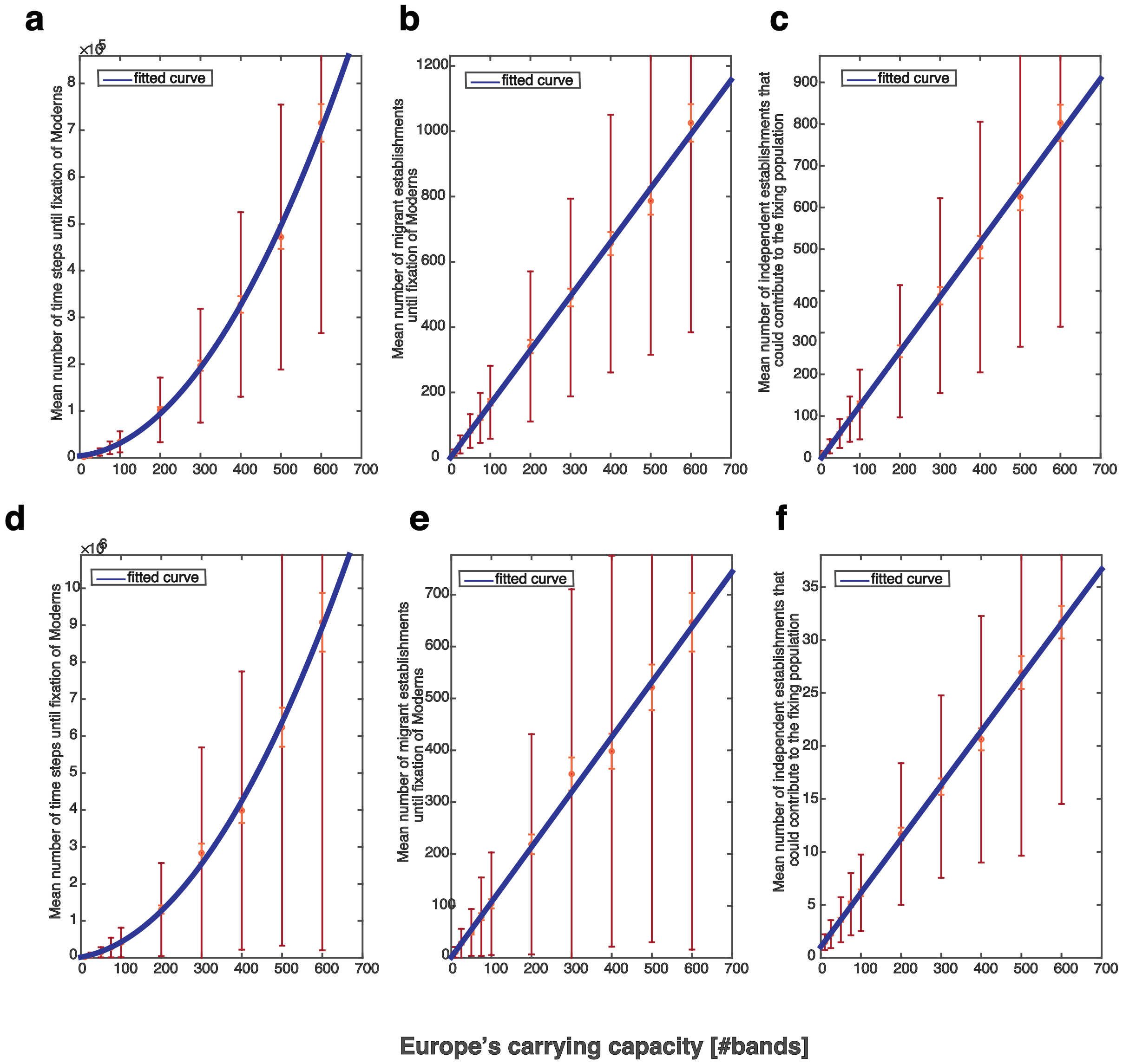

Supplementary Figure 5: The time and number of successful migrations until species replacement under two different migration rates

This figure is analogous to Fig. 1 in the main text, with different migration parameters: in (a)-(c), the probability of migration into Europe per time step is $M_2 = 1$ ($N_2 = 100$, $m_2 = 0.01$). In (d)-(f), the migration rate is lower: $M_2 = 0.05$ (N2 = 100, $m_2 = 0.0005$). (a) and (d) depict the mean number of simulation time steps until complete replacement of Neanderthals by Moderns. The dark blue line is a fit of the means to a quadratic function; the number of time steps to fixation scales with $(N_1)^2$. (b) and (e) depict the mean number of migrant establishments that occur until replacement, which is linearly related to N_1. The dark blue line is a fit to a linear function. (c) and (f) depict the number of migrant establishments that occurred while Moderns were in the population on their way to fixation (see main text). This value scales with N_1. The dark blue line is a fit to a linear function. For all panels, orange error bars denote two standard errors around the mean, dark red bars denote the standard deviation.

Supplementary Note 3: Additional notes on population sizes, population structure, the Levant, and inter-species competition dynamics

Our model assumes, for clarity and parsimony, a very simple demography. We point out a number of demographic considerations that may be important and deserve consideration in future studies.

1. Population sizes: as noted in the main text, the relative population sizes do not affect the determined replacement of Neanderthals by Moderns discussed in the section 'Migration and carrying capacity in unidirectional migration' in the Results; that is, under the assumption of unidirectional migration out of Africa and into the Levant and Europe, which is in line with the archaeological and genetic data to date ('Unidirectional migration from Africa to Europe leads to Neanderthal exclusion' in the Results). The relative population sizes may play a role when bi-directional migration is considered (Figs 4 and 5 in the main text); we find that a difference in initial population sizes between the two species in the two demes has a disproportionate effect on the probability of fixation in favor of the species with the larger initial population. Most accounts suggest a larger Modern population compared to the Neanderthal population, an assessment that is supported most prominently by estimates of diversity in genetic data, combined with archaeological evidence (see, e.g., refs. [24,55–57]), alongside some environmental considerations: based on its size and biological productivity, Africa could have supported a much larger hominid population, and this difference is likely to have been greatly magnified during ice ages in which large parts of Europe were uninhabitable [58,59]. However, some analyses highlight a decrease in Modern populations in Africa near the end of the middle Paleolithic (e.g. ref. [22]), based on the low density of archaeological sites from that period and on reconstruction of climatic conditions at the time, which suggests that large parts of Africa may have been too dry to support hominid populations [60,61]. We remain agnostic regarding this topic.

2. Population substructure: Our model does not take into account population substructure, even though such substructure may definitely play a role in realistically complex dynamics of interspecies interaction. Thus, for example, some would argue that early populations of Moderns in the Levant differ from later out-of-Africa Moderns to the extent that their probable overlap and interaction with Neanderthal populations in the Levant should not be

considered species' co-existence in the duration estimates that we use to assess the validity of our model (as is discussed in Supplementary Note 1) or in deriving support for it based on qualitative features of the two species' time trajectories (as we suggest in the discussion; see, e.g. refs [62,63]. This interpretation is not uncommon, and frequently the two species' long potential overlap in the Levant is not addressed in discussions of their replacement dynamics (e.g. ref. [54]). The present study does not depend on either interpretation; we assess the two species' dynamics under both scenarios. Additional aspects of population substructure may affect our results, and should be considered in future studies, and incorporated into both null models such as ours and into models that hypothesize selective, cultural, or environmental factors in the species' replacement.

3. The Levant: the Levant and Europe are treated in our model as a single deme because most studies and debates have been with respect to within- and out-of-Africa dynamics of Moderns. This simplification is useful and justified in some aspects, but may be misleading or confusing in others, as noted above. The temporal, ecological, behavioral, and evolutionary dynamics of the two species' populations in the Levant, over tens of thousands of years, have been studied extensively, giving rise to important insights, highlighting intriguing questions, and producing a plethora of interesting ideas and hypotheses [53,64,65]. Notable among these have been suggestions that are similar in nature to ours, asserting that a model that invokes neither selective differences nor external factors may be sufficient to explain the two species' interaction in the Levant, and that the end result of these dynamics was a matter of historical contingency [52,66].

We suggest that our simplification of the geography over which the species' dynamics ocurred to a geography of two demes with migration between them is useful, and show that a simple spatially-explicit model gives rise to similar qualitative results. Nonetheless, future modeling may benefit from a more detailed account of the migration process, which treats the Levant as a separate unit from Europe. Similarly, computational models that treat in detail the dynamics within the Levant itself may provide additional insights.

4. Inter-species competition dynamics: our model assumes that interactions between bands are selectively neutral, i.e. that the outcome of competitive interaction is independent of the species' identity of the groups involved, and that the average extent

of competition between any two bands is the same regardless of whether they are of the same species. The possibility that the extent of competition between groups within- and between species was different, and that the species differed in their behavior in this context has been proposed in the past (e.g. refs [67,68]), and is an interesting avenue for future exploration of possible (non-neutral) dynamics. Among others, the species may have differed in their tendency to engage in direct inter-band violence and in the manner in which they did so, they may have differed in their tendency to create alliances with other bands, and they may have differed in their strategic choices in situations of conflict (see, e.g., ref. [69]). Interestingly, it is possible to construct a neutral model that considers alliances among bands in the competition dynamics, if one does not assume that the behavioral rules that govern this behavior differ between the species; this can give rise to interesting dynamics, and may slow down or speed up the replacement process, depending on the assumed underlying regularities that this behavior follows. Since modeling such dynamics requires many assumptions, we did not include them in the current null model. Studying such processes may be a fruitful aspect in future studies.

Supplementary Note 4: The likelihood of finding evidence in the archaeological record for local recurrent species replacement

In order to interpret archaeological findings, such as in the attempt to reconstruct the habitation chronology of a site or a region, it may be insightful to consider the possible authorship of the stone artifacts and their distribution in time. For example, when a certain amount of stone tool artifacts is found at a site, associated with a certain techno-complex and dated to the late middle Paleolithic (e.g. ref. [70]), should it be interpreted as representing the full time range of the site's occupation, sporadic habitation of the site along a number of discrete periods, or perhaps a single short period of habitation within the range of inferred dates associated with the site? More specifically one might ask: how many individual stone knappers produced the assemblage, and how are they related to one another?

Answering this challenging question is well beyond the scope of the current study, but it may be instructive to make a conservative back-of-the-envelope calculation, to get a minimal notion of what we might expect to find in the archaeological record, in order to assess the extent to which the results of our current study's modeling are reasonable. The model suggests that for replacement to have occurred via a neutral process, each band territory would have changed hands a certain average number of times (dependent on the assumed parameter values, see Figures 2 and 6, and Supplementary Figures 1-4). How many of these bands do we expect to have participated in the authorship of a stone tool assemblage that is excavated at a single site? The answer may help tune one's expectation regarding, for example, the likelihood of finding at a single site evidence for recurring species replacement, or of species' co-occurrence.

The figures required for a rough calculation are, at the least, (1) an estimate (to an order of magnitude) of the number of artifacts produced by a single individual, or a single band; and (2) the number of stone artifacts found at a site that are associated with the period of interest, i.e. the layers that date approximately to 55-35kya.

1. The practice required in order to reach a high level of expertise in the production of stone tools characteristic of the technologies of the Middle Paleolithic, and perhaps even some of the Lower Paleolithic, is extensive (see, e.g. refs [71,72], requiring years of

experience. In a very conservative estimate, such experience would entail the production of a few hundreds of stone tools.

2. There is no such thing as “a typical site”, but for our calculation we can consider that the richness of sites in terms of the findings in them that date to the period of interest lie along a spectrum, ranging from those with very few artifacts to those with very many. Naturally, most sites are near the lower end of this spectrum, while only few are at the high end. For our rough calculation it suffices to get an estimate of the order of magnitude of the number of stone artifacts found in the majority of these sites. We estimate that this number is on the order of a few thousand stone tools (see, e.g. refs [70,73–76].

Assuming that each band of hunter-gatherers would have included at least one experienced stone knapper and one or two knappers in their training phase, it becomes obvious that even a single band, over a few generations of habitation, could potentially account for all of the findings at a single archaeological site. A less restrictive approach would suggest that more bands are represented in each such site’s assemblage; increasing this estimate by an order of magnitude leads to an estimate that the authorship of the assemblage at a single site from the period of interest represents ten bands, or perhaps a few dozen in a very permissive estimate. Assuming some variance between bands in the amount of tools left by each, one should expect some of these bands to be represented by a number of tools which is quite small, perhaps so small that its reliable association with a particular techno-complex may be impossible. The timing of the occupation by these bands may be distributed over a time span of thousands of years. Together, these suggest that the archaeological record is extremely sparse in its coverage of the period of interest regarding species’ replacement, i.e. 55-35kya.

Given these (extremely) rough estimates, and even under the (questionable) assumption that bands of different species in Europe of the late Middle Paleolithic are characterized by different stone tool technologies, we suggest that one should not expect to find evidence for contemporaneity nor of recurrent species replacement at any single site, even if replacement of one species by another at each territory occurred multiple times near the transition between the Middle and Upper Paleolithic. This is particularly true when taking into account that the majority of Middle Paleolithic finds, at least in some regions, are from surface

collection (see, e.g., ref [77]), and not excavations of layered sites, thus providing no information about order of replacement and/or contemporaneity.

Supplementary References

1. Müller, U. C. *et al.* The role of climate in the spread of modern humans into Europe. *Quat. Sci. Rev.* **30,** 273–279 (2011).
2. Tzedakis, P. C., Hughen, K. A., Cacho, I. & Harvati, K. Placing late Neanderthals in a climatic context. *Nature* **449,** 206–208 (2007).
3. Green, R. E. *et al.* A draft sequence of the Neandertal genome. *Science* **328,** 710–22 (2010).
4. Valet, J.-P. & Valladas, H. The Laschamp-Mono lake geomagnetic events and the extinction of Neanderthal: a causal link or a coincidence? *Quat. Sci. Rev.* **29,** 3887–3893 (2010).
5. Wolff, H. & Greenwood, A. D. Did viral disease of humans wipe out the Neandertals? *Med. Hypotheses* **75,** 99–105 (2010).
6. Jimenez-Espejo, F. J. *et al.* Climate forcing and Neanderthal extinction in Southern Iberia: insights from a multiproxy marine record. *Quat. Sci. Rev.* **26,** 836–852 (2007).
7. Underdown, S. A potential role for transmissible spongiform encephalopathies in Neanderthal extinction. *Med. Hypotheses* **71,** 4–7 (2008).
8. Hockett, B. & Haws, J. A. Nutritional ecology and the human demography of Neandertal extinction. *Quat. Int.* **137,** 21–34 (2005).
9. Cachel, S. Dietary Shifts and the European Upper Palaeolithic. *Curr. Anthropol.* **38,** (1997).
10. Pike-Tay, A., Valdés, V. C. & de Quirós, F. B. Seasonal variations of the Middle–Upper Paleolithic transition at El Castillo, Cueva Morın and El Pendo (Cantabria, Spain). *J. Hum. Evol.* **36,** 283–317 (1999).
11. Richards, M. P. & Trinkaus, E. Isotopic evidence for the diets of European Neanderthals and early modern humans. *Proc. Natl. Acad. Sci.* **106,** 16034–16039 (2009).
12. Eswaran, V. *et al.* A Diffusion Wave out of Africa: The Mechanism of the Modern Human Revolution? *Curr. Anthropol.* **43,** 749–774 (2002).
13. Trinkaus, E. Neanderthal mortality patterns. *J. Archaeol. Sci.* **22,** 121–142 (1995).
14. Varki, A. Why are there no persisting hybrids of humans with Denisovans, Neanderthals, or anyone else? *Proc. Natl. Acad. Sci.* **113,** (2016).
15. Mellars, P. Neanderthals and the modern human colonization of Europe. *Nature* **432,** 461–465 (2004).
16. Klein, R. G. Whither the Neanderthals? *Science (80).* **299,** 1525 (2003).
17. Gilligan, I. Neanderthal extinction and modern human behaviour: the role of climate change and clothing. *World Archaeol.* **39,** 499–514 (2007).
18. Hortolà, P. & Martínez-Navarro, B. The Quaternary megafaunal extinction and the fate of Neanderthals: An integrative working hypothesis. *Quat. Int.* **295,** 69–72 (2013).
19. Varki, A. Human uniqueness and the denial of death. *Nature* **460,** 684 (2009).
20. Nowell, A. Childhood, play and the evolution of cultural capacity in Neanderthals and modern humans. In *The nature of culture,* eds. Haidle, M.N., Conard, N.J., & Bolus, M. p. 87–97 (Springer, Netherlands, 2016).
21. Gilpin, W., Feldman, M. W. & Aoki, K. An ecocultural model predicts Neanderthal extinction through competition with modern humans. *Proc. Natl. Acad. Sci.* **113,** 2134–2139 (2016).

22. Klein, R. G. Archeology and the evolution of human behavior. *Evol. Anthropol. Issues News Rev.* **9,** 17–36 (2000).
23. Mellars, P. The impossible coincidence: a single species model for the origins of modern human behavior in Europe. *Evol. Anthr.* **14,** (2005).
24. Harris, K. & Nielsen, R. The genetic cost of Neanderthal introgression. *Genetics* **203,** 881–891 (2016).
25. Juric, I., Aeschbacher, S. & Coop, G. The strength of selection against Neanderthal introgression. *PLoS Genet* **12,** e1006340 (2016).
26. Ding, Q., Hu, Y., Xu, S., Wang, J. & Jin, L. Neanderthal introgression at chromosome 3p21.31 was under positive natural selection in East Asians. *Mol. Biol. Evol.* **31,** 683–95 (2014).
27. Abi-Rached, L. *et al.* The shaping of modern human immune systems by multiregional admixture with archaic humans. *Science* **334,** 89–94 (2011).
28. Racimo, F., Sankararaman, S., Nielsen, R. & Huerta-Sánchez, E. Evidence for archaic adaptive introgression in humans. *Nat. Rev. Genet.* **16,** 359–371 (2015).
29. Mendez, F. L., Watkins, J. C. & Hammer, M. F. A haplotype at STAT2 Introgressed from neanderthals and serves as a candidate of positive selection in Papua New Guinea. *Am. J. Hum. Genet.* **91,** 265–74 (2012).
30. Mendez, F. L., Watkins, J. C. & Hammer, M. F. Neandertal origin of genetic variation at the cluster of OAS immunity genes. *Mol. Biol. Evol.* **30,** 798–801 (2013).
31. Belfer-Cohen, A. & Goring-Morris, A. N. From the beginning: Levantine Upper Palaeolithic cultural change and continuity. *Rethinking the Human revolution.* p. 199–206 (University of Cambridge, Cambridge, 2007).
32. Goring-Morris, N. & Davidzon, A. Straight to the point: Upper Paleolithic Ahmarian lithic technology in the Levant. *Anthropologie* **44,** 93 (2006).
33. Teyssandier, N., Bon, F. & Bordes, J.-G. Within projectile range: some thoughts on the appearance of the Aurignacian in Europe. *J. Anthropol. Res.* 209–229 (2010).
34. Bar-Yosef, O. & Belfer-Cohen, A. Following Pleistocene road signs of human dispersals across Eurasia. *Quat. Int.* **285,** 30–43 (2013).
35. Zilhão, J. & d'Errico, F. The chronology of the Aurignacian and Transitional technocomplexes. Where do we stand. in *The chronology of the Aurignacian and of the transitional technocomplexes: dating, stratigraphies, cultural implications. Proceedings of Symposium* **6,** 2–8 (2003).
36. Mellars, P. The Neanderthal problem continued. *Curr. Anthropol.* **40,** 341–364 (1999).
37. Powell, A., Shennan, S. & Thomas, M. G. Late Pleistocene demography and the appearance of modern human behavior. *Science* **324,** 1298–1301 (2009).
38. O'Connell, J. F. & Allen, J. Pre-LGM Sahul (Pleistocene Australia-New Guinea) and the archaeology of early modern humans. *Rethinking the Human Revolution.* p. 395–410 (2007).
39. Brumm, A. & Moore, M. W. Symbolic revolutions and the Australian archaeological record. *Cambridge Archaeol. J.* **15,** 157–175 (2005).
40. James, H. A. & Petraglia, M. Modern Human Origins and the Evolution of Behavior in the Later Pleistocene Record of South Asia. *Curr. Anthropol.* **46,** S3–S27 (2005).
41. Graf, K. E. Modern human colonization of the Siberian mammoth steppe: a view from south-central Siberia. in *Sourcebook of Paleolithic transitions.* Eds. Camps, M. and Chauhan, P. p. 479–501 (New York, Springer, 2009).

42. Kerry, P. J. B. K. W. & Kuzmin, A. I. K. Y. V. Time-Space Dynamics in the Early Upper Paleolithic of Northeast Asia. In *Entering America: Northeast Asia and Beringia Before the Last Glacial Maximum.* Ed. Madsen, D. B. p. 255 (University of Utah Press, 2004).
43. Nigst, P.R., et al. Early modern human settlement of Europe north of the Alps occurred 43,500 years ago in a cold steppe-type environment. *Proceedings of the National Academy of Sciences* 111.40 (2014): 14394-14399.
44. Benazzi, S. *et al.* Early dispersal of modern humans in Europe and implications for Neanderthal behaviour. *Nature* **479,** 525–528 (2011).
45. Mellars, P. Archeology and the dispersal of modern humans in Europe: Deconstructing the 'Aurignacian'. *Evol. Anthropol. Issues, News, Rev.* **15,** 167–182 (2006).
46. Gravina, B., Mellars, P. & Ramsey, C. B. Radiocarbon dating of interstratified Neanderthal and early modern human occupations at the Chatelperronian type-site. *Nature* **438,** 51–6 (2005).
47. Hublin, J. J., Spoor, F., Braun, M., Zonneveld, F. & Condemi, S. A late Neanderthal associated with Upper Palaeolithic artefacts. *Nature* **381,** 224–6 (1996).
48. Hershkovitz, I. *et al.* Levantine cranium from Manot Cave (Israel) foreshadows the first European modern humans. *Nature* **520,** 216–219 (2015).
49. Bar-Yosef, O. Neanderthals and Modern Humans Across Eurasia. in *Dynamics of Learning in Neanderthals and Modern Humans (Volume 1). Eds. Takeru, A., Nishiaki, Y., & Aoki, K.* p. 7–20 (Springer Japan, 2013). doi:10.1007/978-4-431-54511-8_2
50. Higham, T. *et al.* The earliest evidence for anatomically modern humans in northwestern Europe. *Nature* **479,** 521–524 (2011).
51. Pinhasi, R., Higham, T. F. G., Golovanova, L. V & Doronichev, V. B. Revised age of late Neanderthal occupation and the end of the Middle Paleolithic in the northern Caucasus. *Proc. Natl. Acad. Sci. U. S. A.* **108,** 8611–6 (2011).
52. Hovers, E. Neandertals and modern humans in the Middle Paleolithic of the Levant: what kind of interaction? in *When Neanderthals and Modern Humans Met. ed. Conard, N. J.* p. 65–85 (Kerns Verlag, 2006).
53. Shea, J. J. Neandertals, competition, and the origin of modern human behavior in the Levant. *Evol. Anthropol. Issues, News, Rev.* **12,** 173–187 (2003).
54. Higham, T. *et al.* The timing and spatiotemporal patterning of Neanderthal disappearance. *Nature* **512,** 306–309 (2014).
55. Bocquet-Appel, J. P., Demars, P. Y., Noiret, L. & Dobrowsky, D. Estimates of Upper Palaeolithic meta-population size in Europe from archaeological data. *J. Archaeol. Sci.* **32,** 1656–1668 (2005).
56. Bocquet-Appel, J. P. & Degioanni, A. Neanderthal demographic estimates. *Curr. Anthropol.* **54,** S202–S213 (2013).
57. Mellars, P. Why did modern human populations disperse from Africa ca. 60,000 years ago? A new model. *Proc. Natl. Acad. Sci.* **103,** 9381–9386 (2006).
58. Black, B. A., Neely, R. R. & Manga, M. Campanian Ignimbrite volcanism, climate, and the final decline of the Neanderthals. *Geology* **43,** 411–414 (2015).
59. Yustos, P. S. & Martín, F. D. Dancing to the rhythms of the Pleistocene? Early Middle Paleolithic population dynamics in NW Iberia (Duero Basin and Cantabrian Region). *Quat. Sci. Rev.* **121,** 75–88 (2015).

60. Eriksson, A. *et al.* Late Pleistocene climate change and the global expansion of anatomically modern humans. *Proc. Natl. Acad. Sci.* **109,** 16089–16094 (2012).
61. Klein, R. G. & Bird, D. W. Shellfishing and human evolution. *J. Anthropol. Archaeol.* (2016).
62. Klein, R. G. *The human career: human biological and cultural origins*. (University of Chicago Press, 2009).
63. Stringer, C. Palaeoanthropology: coasting out of Africa. *Nature* **405,** 24–27 (2000).
64. Shea, J. J. Neandertal and Early Modern Human Behavioral Variability A Regional-Scale Approach to Lithic Evidence for Hunting in the Levantine Mousterian 1. *Curr. Anthropol.* **39,** S45–S78 (1998).
65. Rak, Y. Morphological variation in Homo neanderthalensis and Homo sapiens in the Levant. in *Species, Species Concepts and Primate Evolution. Eds. Kimbel, W. H., & Martin, L. B.* p. 523–536 (Springer, 1993).
66. Hovers, E. & Belfer-Cohen, A. "Now You See it, Now You Don't"—Modern Human Behavior in the Middle Paleolithic. in *Transitions before the transition. Eds. Hovers, E., & Kuhn, S.* p. 295–304 (Springer, 2006).
67. Gat, A. Social organization, group conflict and the demise of Neanderthals. *Mankind Q.* **39,** 437 (1999).
68. Bingham, P. M. Human evolution and human history: A complete theory. *Evol. Anthropol. Issues News Rev.* **9,** 248–257 (2000).
69. Shea, J. J. The origins of lithic projectile point technology: evidence from Africa, the Levant, and Europe. *J. Archaeol. Sci.* **33,** 823–846 (2006).
70. Malinsky-Buller, A., Ekshtain, R. & Hovers, E. Organization of lithic technology at 'Ein Qashish, a late Middle Paleolithic open-air site in Israel. *Quat. Int.* **331,** 234–247 (2014).
71. Nonaka, T., Bril, B. & Rein, R. How do stone knappers predict and control the outcome of flaking? Implications for understanding early stone tool technology. *J. Hum. Evol.* **59,** 155–167 (2010).
72. Stout, D., Toth, N., Schick, K. & Chaminade, T. Neural correlates of Early Stone Age toolmaking: technology, language and cognition in human evolution. *Philos. Trans. R. Soc. B Biol. Sci.* **363,** 1939–1949 (2008).
73. Ekshtain, R., Barzilai, O., Inbar, M., Milevski, I. & Ullman, M. Givat Rabi East, a new Middle Paleolithic knapping site in the Lower Galilee (Israel). *Paléorient* **37,** 107–122 (2012).
74. Ekshtain, R., Ilani, S., Segal, I. & Hovers, E. Local and Nonlocal Procurement of Raw Material in Amud Cave, Israel: The Complex Mobility of Late Middle Paleolithic Groups. *Geoarchaeology* (2016).
75. Conard, N. J. & Adler, D. S. Lithic reduction and hominid behavior in the Middle Paleolithic of the Rhineland. *J. Anthropol. Res.* **53,** 147–175 (1997).
76. Oron, M. & Goren-Inbar, N. Mousterian intra-site spatial patterning at Quneitra, Golan Heights. *Quat. Int.* **331,** 186–202 (2014).
77. Richter, J. Leave at the height of the party: A critical review of the Middle Paleolithic in Western Central Europe from its beginnings to its rapid decline. *Quat. Int.* **411,** 107–128 (2016).
